# Prevalence and determinants of orthorexia nervosa among Turkish adolescents

**DOI:** 10.1007/s00127-025-02928-x

**Published:** 2025-05-24

**Authors:** Selman Yildirim, Bahadir Turan, Samiye Çilem Bilginer, Esra Hoşoglu

**Affiliations:** 1 Faculty of Medicine, Department of Child and Adolescent Psychiatry, Karadeniz Technical University, Trabzon, Türkiye; 2 Department of Psychology, Avrasya University, Trabzon, Türkiye

**Keywords:** Orthorexia nervosa, Prevalence, Comorbidity, Adolescent mental health, Eating disorders

## Abstract

**Purpose:**

Given the prevalence and diversity of eating issues among adolescents, understanding the epidemiological aspects of orthorexia nervosa (ON) in this demographic, along with identifying associated clinical factors, sociodemographic traits, eating habits and parental attitudes, holds paramount importance both clinically and scientifically. This study aimed to ascertain the prevalence of ON among Turkish high school adolescents aged 14–18 and explore potential correlates of this clinical condition.

**Method:**

In the initial phase, 1784 adolescents completed the Data Form, Orthorexia Nervosa Inventory (ONI) and Parent Style Scale (PSS), while their parents filled out the the parental version of the revised Child Anxiety and Depression Scale (RCADS). Subsequently, clinical interview and The Kiddie-Schedule for Affective Disorders and Schizophrenia for School Aged Children, Present and Lifetime Version (KD-SADS-PL) were conducted with 9 adolescents with high risk for ON to assess comorbidities.

**Results:**

The study revealed ON prevalence of 0.5%, with 0.3% in boys and 0.6% in girls. Rural residency, dietary supplement use, and pet ownership correlated with higher ONI scores (*p* = 0.022, *p* = 0.002, *p* = 0.042 respectively). OCD and panic disorder symptoms (B = 0.307, *p* < 0.001 and B = 0.165, *p* = 0.018 respectively), increased BMI, and anxiety scores were associated with elevated ONI scores. Authoritarian parenting was significantly related to ONI total scores (B = 1.69, *p* = 0.018).

**Conclusion:**

This study significantly contributes to the literature by delineating the prevalence of ON in adolescents, identifying associated risk factors, elucidating psychopathological associations of orthorexic symptoms, and identifying the relationship between parenting styles and ON. Such insights into the factors influencing ON can aid in demystifying its nature.

## Introduction

The term orthorexia was first defined in 1997 by Steven Bratman, based on the Greek words “orthos” meaning “right/correct” and “orexis” meaning “appetite”. Orthorexia Nervosa (ON) is an expression modelled on Anorexia Nervosa to indicate a possible new eating disorder [1]. Unlike Anorexia Nervosa (AN), which is primarily concerned with body weight and food quantity, ON is centered around the perceived quality and purity of food [2, 3]. While ON and AN share common psychological traits such as perfectionism and anxiety, ON does not necessarily involve body image disturbances as a primary feature [4, 5].

ON represents a multifaceted field of study that interweaves psychological, familial, and clinical domains. Research suggests that traits like anxiety, perfectionism, and obsessive-compulsive tendencies may contribute to ON, highlighting potential overlaps with other psychiatric disorders [6, 7]. Additionally, conflicting findings on sociodemographic factors such as age and BMI underscore the need for theoretical models to explain these relationships [8]. Family dynamics are also considered an important factor in the development of ON. Parental attitudes may gently influence children’s dietary habits and their understanding of health, potentially contributing to the development of orthorexic tendencies in adolescents [9, 10]. Understanding ON through both clinical and familial lenses may provide a more comprehensive perspective on its development and risk factors.

Despite the increasing number of studies, many issues such as the prevalence and risk factors of ON remain to be elucidated. This uncertainty may be partly due to the lack of consensus on how ON should be assessed and diagnosed [11–14]. Studies on ON have used various scales and criteria, leading to conflicting results. For instance, the ORTO-15 scale, one of the most commonly used instruments, has yielded varied prevalence rates in adolescent populations. In a study by Hyrnik et al. (2016), different cut-off scores on the ORTO-15 resulted in prevalence rates ranging from 4.2 to 61.3% in a sample of 1899 high school students [15]. Studies with smaller samples in adolescents have reported prevalence rates between 4% and 76.4% [16, 17], further demonstrating the inconsistencies in ON prevalence across different populations and study methods [18].

The discrepancies observed in the summarized literature findings can be largely attributed to limitations in the psychometric properties of the assessment tools employed. The majority of studies have relied on the ORTO-15 and its variations, which are designed to assess orthorexic tendencies but lack the diagnostic specificity required to accurately measure ON. As a result, these tools often produce false positives, especially among individuals with high orthorexic tendencies but without the full symptomatology of ON [19, 20]. The Dusseldorf Orthorexia Scale (DOS) and Orthorexia Nervosa Inventory (ONI) were developed to respond to the need for a reliable assessment of ON. ONI is notable both because of its strong psychometric properties and because it is the only tool that includes an assessment for the criteria of impairment in physical and psychosocial functioning, which are accepted by researchers for the diagnosis of ON [21, 22].

Although only a few studies have been conducted using the Orthorexia Nervosa Inventory (ONI), there is a pressing need for further research to address the gaps and uncertainties surrounding ON. This study aims to fill those gaps by pursuing several key objectives. First, it aims to reliably estimate the prevalence of ON among adolescents using the ONI, which is known for its strong psychometric properties, and ensuring a sample size large enough for accurate prevalence estimation. Second, the study seeks to examine the distribution of psychiatric disorders among adolescents who are at high risk for ON, addressing the lack of detailed understanding of the psychiatric disorders that may coexist. Third, it explores the complex relationships between orthorexic symptoms and common mental health conditions such as depression, anxiety disorders, and obsessive-compulsive disorder (OCD) symptoms, shedding light on the potential psychological factors and relationship of ON. Fourthly, the study aims to investigate the role of parenting attitudes and various potential risk factors in the development of orthorexic symptoms in adolescents, offering a comprehensive perspective on the multifaceted nature of ON.

## Method

### Sampling and participants

This study was conducted by the Department of Child and Adolescent Mental Health and Diseases, Faculty of Medicine, Karadeniz Technical University, as a single-centre and epidemiological study between September 2022 and March 2023 by randomly including students and their families from 17 high schools.

The sample size was determined using the Open Epi programme. For the calculation, a value of 4.5% was used, representing the proportion of orthorexic-prone individuals in the population, based on data from Oberle et al. (2021), the only prevalence study with ONI. A sample size of 1442 was calculated with a deviation of 1.5%, a design effect of 2% due to cluster sampling, and a 95% confidence interval. The 2% design effect was chosen to account for the potential variability introduced by cluster sampling, which can lead to intra-group similarities. It was aimed to recruit at least 1586 participants by adding a 10% margin of error to the calculated sample size [22–24].

The study included literate high school students between the ages of 14 and 18 who were actively enrolled in school and continuing their education, and for whom the approval of the school administration and the written informed consent of both the adolescent and the parent were obtained. Adolescents and parents who either refused to participate or withdrew at any stage of the study, as well as those with incomplete questionnaires, were excluded. We also excluded participants with significant cognitive impairments or medical conditions that, according to the assessment of the child psychiatrist who distributed the questionnaires and information obtained from the relevant teacher, might interfere with completing the assessments to ensure data validity.

### Procedure

Ethical approval for the study was obtained from Karadeniz Technical University Faculty of Medicine Scientific Research Ethics Committee (number: 2022 − 127, date: 30.06.2022) and permissions were acquired from Trabzon Governorship Provincial Directorate of National Education for data collection from high schools. No personal data of the participants were used in the study.

The study was conducted in two stages. In the first stage, 17 high schools were randomly selected by the researchers from a total of 145 high schools in Trabzon province. Each questionnaire was distributed to an equal number of high school 1st, 2nd, 3rd and 4th grades from the selected schools. I In the first phase, a total of 3450 students were distributed the Data Form, Orthorexia Nervosa Inventory (ONI) and Parenting Style Scale (PSS), and literate parents were invited to complete the parent version of the Revised Child Anxiety and Depression Scale (RCADS). 1666 participants were excluded from the study due to incomplete questionnaires or because the parents or participants did not give consent to participate. A total of 1784 students whose consent was obtained and who completed the questionnaires, were included in the study. In the second stage, The Kiddie-Schedule for Affective Disorders and Schizophrenia for School Aged Children, Present and Lifetime Version (KD-SADS-PL) were conducted with 9 participants with high risk for ON, who scored 72 points or more according to the ONI.

In the second stage, both DSM-5-based clinical interviews and Kiddie-Schedule for Affective Disorders and Schizophrenia for School-Aged Children, Present and Lifetime Version (KD-SADS-PL) semi-structured interviews were applied to 9 participants with high risk for ON, who scored 72 points or more according to the ONI. The choice of the 72-point cut-off for the ONI was based on previous study by Oberle et al. (2021), who used this threshold to identify individuals at high risk for ON [22]. The clinical interviews were conducted by a trained child psychiatrist, to assess the psychiatric diagnoses of the participants with high risk for ON.

### Measures

#### Data form

This form consists of questions describing the sociodemographic characteristics of the sample (Gender, place of living, family type, family income), medical condition (Psychiatric disorder, chronic medical disease and history of Covid-19 in the past 6 months) and nutritional behaviour and lifestyle that may be related to ON (Body mass index, following a diet programme, preferred nutritional style, use of dietary supplements as multivitamins, the way of consuming meals, pet ownership, daily social media duration and weekly exercise frequency) obtained by the researcher by reviewing the literature on ON and other eating disorders based on the fact that ON is a possible eating disorder (Table 1) [8, 14, 25, 26]. Family income status was evaluated based on the poverty line in Türkiye at the time the questionnaire was distributed [27]. The family income information was provided by the parents.

#### Parenting style scale (PSS) [28]

The scale was developed by Lamborn et al. (1991). Multivariate analysis of covariance (MANCOVA) conducted by the researchers on the scale scores revealed three dimensions: Acceptance/Involvement, Strictness/Supervision, and Psychological Autonomy. According to the proposed scoring method, the parents who scored above the median in the acceptance/involvement and strictness/supervision dimension were categorised as ‘democratic’ and those who scored below the median were categorised as ‘permissive-neglectful’. Also, the parents of the participants who scored below the median on the acceptance/involvement dimension and above the median on the strictness/supervision dimension were labelled as ‘authoritarian’, and the parents of the participants who scored above the median on the acceptance/interest dimension and below the median on the supervision dimension were labelled as ‘permissive-tolerant’. The Cronbach’s alpha coefficients for the dimensions were as follows: Acceptance/Involvement: 0.72, Strictness/Supervision: 0.76 and Psychological Autonomy: 0.82 in the original study [28]. The Turkish validity and reliability of the scale was conducted by Yılmaz (2000). For Turkish high school students, the reliability coefficients and internal consistency coefficients were found as follows: acceptance/involvement subscale: 0.82 and 0.70, strictness/supervision subscale: 0.88 and 0.69, and psychological autonomy subscale: 0.76 and 0.66 [29].

#### The revised child anxiety and depression scale – parent version (RCADS-Parent) [30]

It was developed by Chorpita et al. to measure DSM-IV based anxiety disorders and depression symptoms. The scale consists of 47 items and has a 4-point likert type. Turkish validity and reliability study was conducted by Gormez et al. (2017). According to the scoring, subscale scores of Generalised Anxiety Disorder, Social Phobia, Separation Anxiety Disorder, Panic Disorder, Obsessive Compulsive Disorder and Major Depressive Disorder are obtained. In addition, total anxiety and total internalising disorder scores can also be calculated as a result of the evaluation of the scale [31].

#### Orthorexia nervosa inventory (ONI) [22]

It was developed by Oberle et al. (2021) to evaluate ON. The 4-point Likert-type scale consists of 24 items. Cronbach’s alpha internal consistency coefficient was found to be 0.94 in development study. It has three subscales including behaviour, impairment in physical and psychosocial functioning and emotional stress. As the total scale score and subscale scores increase, symptom severity also increases. Above the cut-off score of 72 determined for the scale means ON or high risk in terms of ON. Turkish validity and reliability are available in adults and adolescents. The Cronbach’s alpha coefficient was 0.92 in Turkish adolescents and 0.91 in Turkish adults [32, 33].

#### The kiddie-schedule for affective disorders and schizophrenia for school aged children, present and lifetime version (KD-SADS-PL) [34]

KD-SADS-PL is a semi-structured interview form. This clinical interview form was prepared by Kaufman et al. (1997) according to DSM-III-R and DSM-IV diagnostic criteria to screen psychopathologies in children and adolescents aged 6–18 years. The current version of the form according to DSM-5 diagnostic criteria was translated into Turkish by Unal et al. (2016) [35].

### Statistical analysis

Statistical analyses were performed using version 23 of SPSS software. Student T test was applied in the analyses of two-group independent variables that fit the normal distribution. Mann Whitney U test was used in the analysis of independent variables with two groups that did not fit the normal distribution, and Kruskal Wallis test was used in the analysis of independent variables with more than two groups. When a significant difference was found between independent variables with more than two groups as a result of Kruskal Wallis test, post-hoc analyses (Tamhane’s T2) were applied to determine between which groups there was a statistically significant difference.

The effects of quantitative variables on ONI scores were analysed by creating multiple linear regression models using the enter method. This was updated by performing two different analyses: one with the subscale scores of the RCADS and the other with body mass index and total anxiety scores. Also, dummy variables were created to include democratic, permissive-neglectful, authoritarian and permissive-tolerant parenting types obtained from the PSS scale in the regression model. In the regression models regarding the predictive effect of parenting types on ONI scale scores, one of the dummy variables was not included in the model by accepting it as reference and multicollinearity was prevented. In regression analyses, model fit was evaluated using residual data and fit statistics. In statistical analyses, *p* < 0.05 was accepted as significance value.

## Results

The mean age of the participants was 15.31 ± 1.10, the mean height was 166.06 ± 8.51, the mean weight was 59.03 ± 12.29 and the mean body mass index was 21.33 ± 3.62. The characteristics of the sample are presented in Table 1.

**Table 1:** Characteristics of the sample

*n* = 1784		Number (%)
Gender	Boy	680 (38.1)
	Girl	1104 (61.9)
Place of Living	Urban	807 (46.4)
	Rural	934 (53.6)
Family Type	Nuclear	1476 (85.4)
	Extended	183 (10.6)
	Fragmented	70 (4.0)
Family İncome	Below the Poverty Line	765 (43.8)
	Above the Poverty Line	983 (56.2)
Psychiatric Disorder	Yes	73 (4.1)
	No	1694 (95.9)
Chronic Medical Disease	Yes	163 (9.3)
	No	1587 (90.7)
Following a Diet Programme	Yes	60 (3.4)
	No	1716 (96.6)
Preferred Nutritional Style	Yes	74 (4.2)
	No	1704 (95.8)
Use of Dietary Supplements	Yes	249 (14.1)
	No	1523 (85.9)
The Way of Consuming Meals	With Family	1255 (71.2)
	With Friends	162 (9.2)
	Alone	345 (19.6)
Pet Ownership	Yes	398 (22.3)
	No	1383 (77.7)
COVID-19 in the Past 6 Months	Yes	197 (11.1)
	No	1577 (88.9)
Daily Social Media Duration	0–2 h	722 (41.0)
	2–4 h	725 (41.1)
	More than 4 h	316 (17.9)
Weekly Exercise Frequency	I never do	669 (37.6)
	0–2 days	822 (46.3)
	More than 2 days	286 (16.1)

It was found that the total prevalence of ON was 0.5%. The prevalence of ON was found to be 0.3% in boys and 0.6% in girls.

As a result of the clinical structured interview and KD-SADS-PL conducted on 9 adolescents with high risk for ON, 5 participants had comorbid eating disorders (4 Bulimia Nervosa and 1 Anorexia Nervosa). The other psychiatric disorders of these participants were Anxiety Disorders in 3 participants, Attention Deficit and Hyperactivity Disorder in 2 participants, Major Depression in 2 participants.

Table 2 presents the analysis of demographic and lifestyle factors selected for their potential relevance to eating behaviours and nutritional habits, including gender, place of living, family income, chronic medical disease, use of dietary supplements, and pet ownership. Those who lived in rural areas (*p* = 0.022), those using dietary supplements (*p* = 0.002) and those owning pets (*p* = 0.042) had significantly higher ONI total scores than the other groups (Table 2).

**Table 2:** ONI total scale scores and the characteristics of the participants

ONI Scale Total Score				
Variables		Mean ± SD	Analysis	*p*
Gender	Boy	32.25 ± 8.09	*Z=-1.11*	*0.269*
	Girl	32.82 ± 8.88		
Place of Living	Urban Area	32.25 ± 8.85	*Z=-2.30*	***0.022***
	Rural Area	32.89 ± 8.41		
Family İncome	Below the Poverty Line	32.91 ± 8.82	*Z=-0.90*	*0.368*
	Above the Poverty Line	32.41 ± 8.52		
Chronic Medical Disease	Yes	34.52 ± 11.02	*Z=-1.82*	*0.069*
	No	32.44 ± 8.32		
Use of Dietary Supplements	Yes	34.73 ± 10.52	*Z=-3.04*	***0.002***
	No	32.26 ± 8.21		
Pet Ownership	Yes	33.17 ± 8.51	*Z=-2.03*	***0.042***
	No	32.44 ± 8.62		

Z = Mann Whitney U test

It was demonstrated that the score of the OCD subscale (B = 0.307, *p* < 0.001) and the score of the panic disorder subscale (B = 0.165, *p* = 0.018) were significantly associated with the ONI total scale score. It was found that an increase in BMI and the total anxiety score of the RCADS were significantly related to higher ONI total scale scores (Table 3). Additionally, authoritarian parenting type was more strongly associated with the ONI total score than other parenting types (B = 1.69, *p* = 0.018) (Table 4).

**Table 3:** Multivariate linear regression analysis of various determinants of ON

	ONI Total		
	B	CI	*p*
Social Phobia	0.041	-0.06-0.14	0.426
Panic Disorder	0.165	0.03–0.30	**0.018**
Major Depression	0.037	-0.07-0.14	0.480
Separation Anxiety Disorder	0.134	-0.05-0.32	0.154
Generalised Anxiety Disorder	0.038	-0.14-0.22	0.672
Obsessive Compulsive Disorder	0.307	0.14–0.48	**< 0.001**
Explanation of the Model (Adjusted R^2^)	0.066		
Significance (p)	**< 0.001**		
Body Mass İndex	0.449	0.34–0.56	**< 0.001**
Total Anxiety	0.132	0.11–0.16	**< 0.001**
Explanation of the Model (Adjusted R^2^)	0.096		
Significance (p)	**< 0.001**		

Multiple Linear Regression Analysis, Enter Method

**Table 4:** Multivariate linear regression analysis of the predictive effect of parenting types on ON

	ONI Total		
	B	CI	*p*
	32.01	31.05–32.97	**0.001<**
Democratic	0.309	-0.91-1.53	0.620
Permissive Neglectful	1.24	-0.05-2.53	0.060
Authoritarian	1.69	0.29–3.09	**0.018**
Permissive Tolerant			
Explanation of the Model (Adjusted R^2^)	0.004		
Significance (p)	**0.043**		

Multiple Linear Regression Analysis, Enter Method

## Discussion

The most striking finding of this study was that the prevalence of ON in adolescents was found to be 0.5%. In studies conducted in adolescents in Turkiye, the prevalence of ON ranged between 1.9% and 76.4% [16, 17, 36, 37]. In the only study conducted in countries other than Turkiye including adolescents, the prevalence of ON was reported as 30.7% [38]. Although there are ON prevalence studies in wider age ranges that evaluate both adolescents and adults together, studies including only adolescents are limited. Considering that adolescents have different developmental and social characteristics compared to adults, it can be said that studies carried out in a more limited age range as in our study may better reflect the prevalence of ON in adolescents.

The relatively lower prevalence rate observed in our study may be attributed to the characteristics of the assessment tool used. Unlike other commonly used instruments, the ONI has demonstrated strong validity and reliability and is designed to evaluate not only orthorexic tendencies but also the psychological impairment associated with them. Since the ONI considers Orthorexia Nervosa as a potential disorder rather than merely an eating behavior tendency, it is likely to provide a more accurate estimation of true ON prevalence in adolescents [18, 22].

In our study, no significant gender differences were found in orthorexic symptoms, although the prevalence of ON was twice as high in girls. Previous literature, mainly based on adult samples, often uses tools with questionable psychometric properties, which may not capture gender differences effectively [8]. The higher prevalence in girls could be linked to societal pressures on body image and aesthetic appearance, which are more prominent in females. Boys, on the other hand, may be more focused on achieving a muscular physique, leading to restrictive eating patterns for different reasons. These factors suggest that while the motivations for orthorexic behaviors may differ by gender, both boys and girls may engage in similar behaviors influenced by societal and cultural factors [39].

In our study, it was an interesting result that the orthorexic symptoms were higher in adolescents living in rural areas. Our study provided the first data on the relationship between ON and living in urban and rural areas. In a recent study, it was reported that the frequency of general psychopathology was higher in children living in rural areas compared to children living in other settlements [40]. Moreover, the easier access of people in rural areas to natural foods without additives may have played a role in the higher orthorexic symptoms [41]. It is likely that dietary supplements are used to preserve personal wellness in ON individuals, which is generally perceived as harmless and pure [42].

Our study found a link between higher BMI and orthorexic symptoms, while the literature shows mixed findings. No studies have explored this in adolescents, where BMI increase may trigger orthorexic behaviors through diet-related concerns. The relationship likely depends on sample characteristics and how ON is conceptualized [8, 36].

Consistent with the studies in the literature, it was found that having authoritarian parents predicted higher orthorexic symptoms in adolescents [43, 44]. Parenting styles can significantly influence the development of ON, with authoritarian parenting potentially creating an environment where emotional needs are not adequately met. While authoritarian parents tend to enforce strict rules and high expectations, they may provide limited emotional support, which can contribute to the development of maladaptive coping mechanisms, such as an excessive focus on food and body image. This may predispose adolescents to orthorexic tendencies, where control over food becomes a way to manage unmet emotional needs. Also, these findings align with psychoanalytic interpretations of eating disorders, which suggest that early emotional neglect or criticism may contribute to the development of rigid behaviors around food [45, 46]. This study emphasizes the importance of considering family dynamics, particularly parenting style, in understanding the development of ON and suggests that intervention programs targeting emotional support and family interactions may help reduce orthorexic behaviors in adolescents.

There are no prevailing views in the literature on the possible associations between orthorexic symptoms and OCD, depression and anxiety disorders. In the current study, OCD symptoms and anxiety disorder symptoms predicted orthorexic symptoms, while depressive symptoms did not. In a multicentre study, it was found that orthorexic symptoms was higher in patients with OCD compared to the control group [47] while Costa et al. (2018) reported that participants with obsessions had fewer orthorexic symptoms [48]. In a recent meta-analysis study, ON symptoms were reported to be more associated with eating disorders compared to OCD [14]. Although ON and OCD have similar symptomatology, it is necessary to examine whether the possible relationship is based on common neurobiological and genetic mechanisms. In a recent prospective study, higher orthorexic symptoms at baseline predicted an increase in depressive symptoms in the evaluation performed 3 months later [49]. Although depression is not a predictor for the orthorexic symptoms, it may precipitate the emergence of depressive symptoms due to the nature of orthorexic symptoms. In two recent studies, it was reported that patients with somatoform disorders showed more orthorexic symptoms [50, 51]. It is considered that increased anxiety symptoms in individuals with more orthorexic symptoms may arise from disease anxiety and the idea of improving personal health, which is assumed to be one of the main motivations of ON, may be triggered by disease anxiety [51].

This study provides valuable insights into the prevalence and predictors of Orthorexia Nervosa (ON) in adolescents, with a key strength being the use of a validated and reliable instrument, the ONI, to assess both orthorexic tendencies and related psychological impairments. Our findings contribute to the literature by highlighting unique factors such as the association between rural living, BMI, and parenting style with orthorexic symptoms. These findings have important clinical implications, particularly in recognizing high-risk groups, such as adolescents from rural areas or those with authoritarian parenting, for early intervention.

Despite the valuable contributions of this study, there are several limitations that should be considered. The assessment of ON prevalence in a cross-sectional design is considered a limitation. Evaluating the prevalence of ON, which is conceptualised as a possible psychiatric disorder, with longitudinal cohort studies may contribute to the literature. Moreover, since the number of participants with ON was very low, no comparison could be performed by forming two separate groups. This limitation can be minimised in future studies with a study design in which high-risk individuals in terms of ON in the clinical sample are compared with the control group. Another limitation of our study is the lack of further analyses on eating disorders. Sufficient inferences could not be made about the possible relationship between eating disorders and ON. In future studies, eating disorders can be subdivided into subtypes and analyses on the possible relationship between eating disorders and ON symptoms can be performed. Additionally, the R² value in the model used in this study was relatively low, indicating that the model explained only a portion of the variability in the data. This suggests that there may be other important variables not included in the model that could better explain the relationship between the predictors and ON. Future studies could explore additional factors, potentially improving the model fit and the explanatory power.

Our study stands as a pioneering investigation into Orthorexia Nervosa (ON), shedding light on its intricacies among adolescents. With this comprehensive understanding of ON, both clinical and scientific breakthroughs appear attainable in the foreseeable future. Ultimately, elucidating the mechanisms underpinning the transition from subthreshold ON symptoms to full-fledged ON in individuals with risk factors emerges as a pivotal pursuit for forthcoming studies.

## Data Availability

No datasets were generated or analysed during the current study.
